# Effect of prolonged exposure to diesel engine exhaust on proinflammatory markers in different regions of the rat brain

**DOI:** 10.1186/1743-8977-7-12

**Published:** 2010-05-17

**Authors:** Miriam E Gerlofs-Nijland, Damien van Berlo, Flemming R Cassee, Roel PF Schins, Kate Wang, Arezoo Campbell

**Affiliations:** 1 Centre for Environmental Health, National Institute for Public Health and the Environment (RIVM), Bilthoven, the Netherlands; 2 Institut für umweltmedizinische Forschung (IUF) at the Heinrich-Heine-University, Düsseldorf, Germany; 3 Pharmaceutical Sciences, College of Pharmacy, Western University of Health Sciences, 309 E. Second Street, Pomona, California 91766-1854, USA

## Abstract

**Background:**

The etiology and progression of neurodegenerative disorders depends on the interactions between a variety of factors including: aging, environmental exposures, and genetic susceptibility factors. Enhancement of proinflammatory events appears to be a common link in different neurological impairments, including Alzheimer's disease, Parkinson's disease, amyotrophic lateral sclerosis, and multiple sclerosis. Studies have shown a link between exposure to particulate matter (PM), present in air pollution, and enhancement of central nervous system proinflammatory markers. In the present study, the association between exposure to air pollution (AP), derived from a specific source (diesel engine), and neuroinflammation was investigated. To elucidate whether specific regions of the brain are more susceptible to exposure to diesel-derived AP, various loci of the brain were separately analyzed. Rats were exposed for 6 hrs a day, 5 days a week, for 4 weeks to diesel engine exhaust (DEE) using a nose-only exposure chamber. The day after the final exposure, the brain was dissected into the following regions: cerebellum, frontal cortex, hippocampus, olfactory bulb and tubercles, and the striatum.

**Results:**

Baseline levels of the pro-inflammatory cytokines tumor necrosis factor alpha (TNF-α) and interleukin-1 alpha (IL-1α) were dependent on the region analyzed and increased in the striatum after exposure to DEE. In addition, baseline level of activation of the transcription factors (NF-κB) and (AP-1) was also region dependent but the levels were not significantly altered after exposure to DEE. A similar, though not significant, trend was seen with the mRNA expression levels of TNF-α and TNF Receptor-subtype I (TNF-RI).

**Conclusions:**

Our results indicate that different brain regions may be uniquely responsive to changes induced by exposure to DEE. This study once more underscores the role of neuroinflammation in response to ambient air pollution, however, it is valuable to assess if and to what extent the observed changes may impact the normal function and cellular integrity of unique brain regions.

## Background

There is an association between chronic exposure to combustion-related fine particles (present in air pollution) and an increased risk of mortality attributed to lung cancer and cardiopulmonary causes [1]. One of the major contributors to particulate air pollution is diesel engine exhaust. As diesel fuel undergoes combustion in automobile engines, it produces particles of different sizes, chemical composition, and physical characteristics [2]. Because of the substantial number of epidemiological studies showing a link between exposure to air pollution and adverse cardiovascular changes, the need for air quality controls and suggestions for future research has been addressed [3]. Recent studies show that the cardiopulmonary system may not be the only vulnerable target adversely affected by air pollution. The brain may be another potential target [4,5].

In a prospective birth cohort study, the association between exposure to black carbon, which is a surrogate for traffic-related particles, and cognition among children was assessed. The authors discovered that higher exposure to black carbon was associated with a decline in cognitive function [6]. Children may not be the only sensitive subpopulation since mild cognitive impairment, associated with long-term exposure to traffic-related particulate air pollution, was also identified in elderly women [7]. These observed changes in cognitive function may be related to alteration in brain activity. Indeed, in human volunteers, brain activity assessed by quantitative electroencephalography, was altered after short-term exposure to diesel engine exhaust [8]. Although it is not known exactly how exposure to ambient air pollution or more specifically, diesel engine exhaust, may alter human brain function, studies indicate that neuroinflammation may play a role.

One of the first studies which pointed towards the potential adverse central nervous system (CNS) effects of air pollution showed that in dogs living in polluted areas of Mexico City, there is an increase in cerebral inflammation [9]. In a later study, using human post-mortem tissue, it was discovered that markers of both neuroinflammation and Alzheimer's disease (AD) pathology are increased in residents living in high (compared to low) pollution cities [10]. In a recent study, the same investigators have validated their earlier findings by showing enhanced neuroinflammation, altered blood-brain barrier characteristics, and particulate deposition in the brain of children and young adults living in cities with high air pollution [11]. In addition, a few toxicological studies in rodents have been published that support these findings. Exposure to ambient particles, present in urban air pollution, enhances neuroinflammatory markers in the brain of mouse models [4,12-14].

In the present study, rats were exposed to diesel engine exhaust (a major contributor to ambient air pollution) to examine neuroinflammatory changes in different brain regions. We hypothesized that the accompanying neuroinflammatory response will be variable in specific brain areas.

## Materials and methods

### Animals

Male Fischer F344/DUCRL rats (15-16 wks old) were obtained from Charles River (Sulzfeld, Germany). The rats were randomly allocated to either control or DEE exposure group (n = 15/group) and acclimatized for 7 days. Experiments were approved by the Animal Ethics Committee (IUCAC) of the Dutch National Vaccine Institute (NVI, Bilthoven, Netherlands).

### Exposure and characterization of test atmosphere

All animals were exposed in whole body inhalation chambers to 0.4 ppm ozone for 12 hrs before initiating the diesel engine exhaust (DEE) exposures. The work described in this paper is an extension of another study aimed at evaluating the effect of prolonged exposure to traffic related particulate matter (PM) on the cardiopulmonary system. These cardiopulmonary effects were studied in the presence of a mild inflammation status induced by ozone [15]. Nose-only exposure to DEE began the next day and continued for 4 wks, 5 days/wk for 6 hrs a day. Control animals were exposed to conditioned, purified and HEPA filtered air with the same temperature and relative humidity as the test atmosphere. Diluted DEE was obtained by mixing freshly generated exhaust from an idling diesel engine (35 KVA Genset, 1500 rpm) with purified air. Particle number and mass concentration were measured continuously in the inlet of the exposure chamber using a condensation particle counter (CPC model 3022A, TSI St. Paul, Minn., USA) or a nephelometer (DATARAM 2000, MIE, Billerica, Mass., USA) respectively. Time-integrated particle concentrations were determined by gravimetric analysis. A carbon sampler tube was placed downstream of one of the PolyTetraFluoroEthylene filters at the outlet to collect the volatile organic compounds (VOC), which were measured by means of GC-MS. The final particle mass concentration in diluted DEE was 173 μg/m^3 ^with a geometric median diameter of 76 nm and geometric standard deviation of 5 nm. Total VOC content was 529 μg/m^3^. The concentrations of CO, NO, and NO_2_, NOx measured in the mixing chamber were on average 2.6 ppm CO, 0.08 ppm NO, 1.3 ppm NO_2 _and 0.5 ppm NOx. These are levels that people will experience in tunnels, workplace or at traffic hot spots.

### Necropsy

Animals were sacrificed 24 hrs after the last exposure. The brain of 10 animals per group was carefully excised and the following regions were dissected on ice: olfactory bulbs and tubercles, striatum, hippocampus, cortex, and the cerebellum. A subset of these samples (n = 5) was used for the ELISA and gel shift mobility assays and another subset (n = 5) was used for the RNA isolation and quantitative PCR analysis.

### Sample preparation for ELISA and gel shift mobility assays

Cytoplasmic and nuclear protein fractions were prepared using the method of Lahiri and Ge [16]. Tissue was weighed and homogenized in ice-cold buffer consisting of (10 mM HEPES (pH 7.9), 10 mM KCl, 0.1 mM EDTA, 0.1 mM EGTA, 1 mM DTT, 0.5 mM PMSF and 0.5% NP-40). The suspension was incubated on ice for 10 min and centrifuged (4000 × g) at 4°C for 1 min. The supernatant containing the cytoplasmic constituents was collected and protease inhibitor was added. Aliquots of samples were stored at -80°C. The nuclear pellet was resuspended in a buffer composed of (20 mM HEPES pH 7.9, 400 mM NaCl, 1 mM DTT, 1 mM EDTA, 1 mM EGTA and 1 mM PMSF). The samples were then centrifuged (11,000 × g) for 5 min at 4°C. The supernatant (which is the nuclear extract) was collected, protease inhibitor was added, and aliquots were prepared and stored at -80°C.

### Competitive Enzyme Immunoassay

Levels of TNF-α and IL-1α were determined using immunoassay kits from Biosource (Camarillo CA), for the detection of the total protein in the cytoplasmic tissue fractions. Briefly, 50 μl of the sample was added to plates precoated with an antibody specific for either rat TNF-α or rat IL-1α. After the addition of a Biotinylated secondary antibody, the plates were washed and incubated with streptavidin-Peroxidase. After another wash, a substrate solution was added and the color generated was determined with a spectrophotometric plate reader set at 450 nm.

### Electrophoretic Mobility Shift Assay

This assay was utilized to determine the extent of NF-κB and AP-1 activation in the nuclear fractions using a protocol developed by Promega (Madison WI). The amount of protein in 2 μl of the nuclear extract was determined by the BCA protein assay kit (Pierce, Rockford, IL) and 25 μg of each sample, incubated with ^32^P-labeled oligonucleotides containing the NF-κB or AP-1 consensus sequence, was loaded onto a gel. A negative control containing no cell extract, as well as competitor reactions were run simultaneously with the samples. The specific competitor contained unlabelled NF-κB or AP-1 consensus nucleotide while the nonspecific competitor contained unlabelled SP-1 consensus oligonucleotide. The competitor reactions also contained 1 g of HELA cell extract (positive control). X-ray films were manually developed. The mean intensity of each band was measured and quantitated using a Kodak 1500 gel logic imaging system.

### Analysis of mRNA

Total RNA was isolated from the various brain tissue regions using the TRIzol Plus RNA Purification Kit (Invitrogen, Carlsbad, CA). The mRNA levels of TNF-α and TNF-RI were determined by quantitative RT-PCR. RNA was purified using the RNeasy^® ^mini kit coupled to treatment with DNase (RNAse-free DNAse set, Qiagen). The purity and amount obtained were determined by spectrophotometry at wave lengths of 230, 260, 280, and 320 nm. From 0.5 μg RNA, cDNA was synthesized using the iScript cDNA Synthesis kit (BioRad, CA, USA) and used for qRT-PCR at a 15 × dilution in RNAse-free water. PCR primers for TNF-α, TNF-RI and the housekeeping gene HPRT were designed using Primer express software (Applied Biosystems). Real time PCR was performed employing a MyIQ Single Color real time PCR detection system (BioRad) coupled to SYBR^© ^Green Supermix (Biorad), added to the system along with 15 × diluted cDNA and 0.3 μM forward and reverse primers. During the PCR reaction, a denaturation step at 95°C for 3 min was followed by 40 cycles at 95°C (15 seconds) and 60°C (45 seconds). To ascertain that the correct product was amplified, a melt curve (60-95°C) was produced. The efficiencies of all primer sets were tested by the generation of cDNA dilution curves. Obtained data was analyzed using MyIQ software (BioRad) and expressed as fold increase compared to the lowest expression in non-treated controls [17].

### Statistical Analysis

Differences between the exposure groups were tested using analysis of variance (ANOVA). Pairwise comparisons were tested using the Tukey method.

## Results

### Cytokine Levels

**Figure 1:**
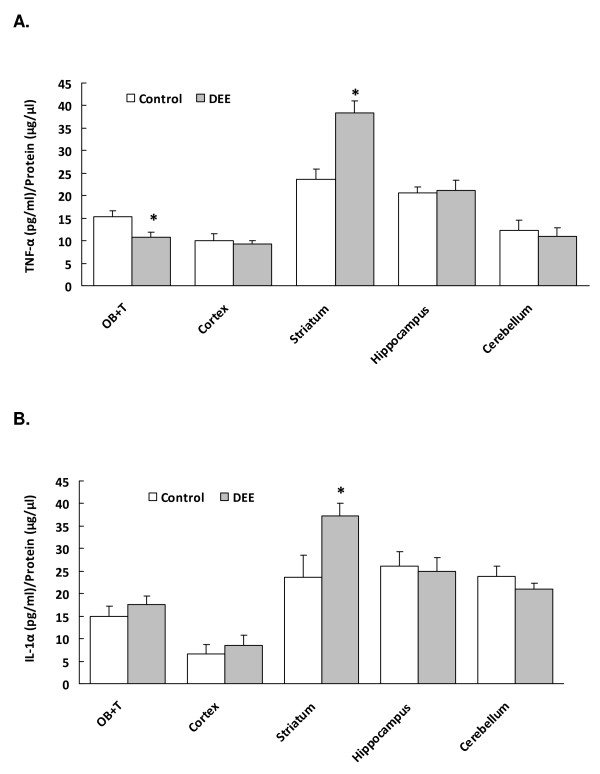
**Pro-inflammatory cytokine levels**. **A**. Levels of TNF-α in different regions of the rat brain after exposure to filtered air (Control) or DEE (Diesel-exposed). Values given as mean ± SE; n = 5; *p < 0.05 compared to control. OB+T = olfactory bulb and the tubercles. **B**. Levels of IL-1α in different regions of the rat brain after exposure to purified air (Control) or DEE (Diesel-exposed). Values given as mean ± SE; n = 5; *p < 0.05 compared to control. OB+T = olfactory bulb and the tubercles.

Basal levels for both TNF-α and IL-1α were detected in all brain regions and varied depending on the specific area analyzed (Figure 1). The levels of the proinflammatory cytokines TNF-α and IL-1α were measured in the cytoplasmic fractions derived from the following brain regions: olfactory bulb and the tubercles (OB+T), cortex, striatum, hippocampus, and cerebellum. The levels of TNF-α were slightly (but significantly) decreased in the OB+T after exposure to DEE, but there were no changes in the levels of IL-1α in this region. However, in the striatum, there was a pronounced increase in the levels of both proinflammatory cytokines. There were no changes in the levels of these two proinflammatory cytokines in the cortex, hippocampus, or cerebellum, after exposure to DEE.

### Transcription factor activation

**Figure 2:**
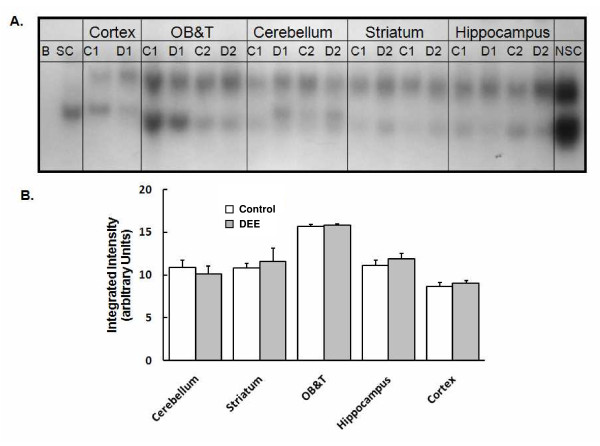
**NF-κB activation after DEE exposure**. **A**. A gel demonstrating NF-κB shifted bands. Samples from different brain regions were assayed on the same gel. Based on the competitor reactions, the top band is specific for NF-κB. B = Blank; SC = Specific competitor; NSC = Non-specific competitor; C = nuclear fraction derived from the brain of animals exposed to filtered air; D = nuclear fractions derived from the brain of animals exposed to DEE; the results for two separate animals (designated as 1 or 2) are shown on this gel. **B**. The sum intensity of NF-κB specific shifted band (first band shown above) in different regions of the rat brain after exposure to purified air (Control) or DEE (Diesel). Each brain region was analyzed on different days on separate gels and therefore this figure does not allow direct comparison between various brain regions. Bars represent mean of 5 individual animals ± SE.

**Figure 3:**
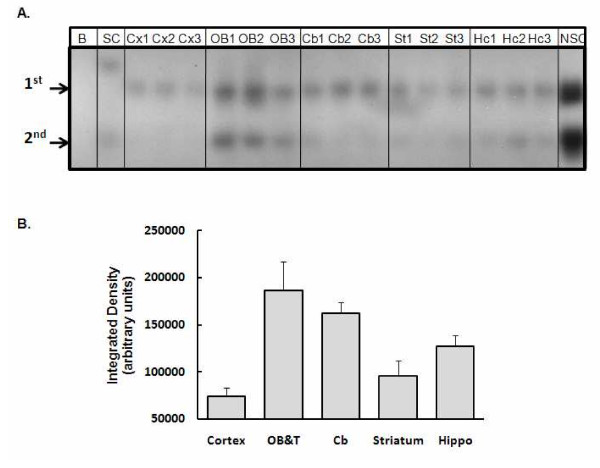
**Basal levels of NF-κB activation**. A. A typical gel showing the NF-κB specific shifted bands for three separate control samples (designated as 1, 2, or 3). B = Blank; SC = Specific competitor; NSC = Non-specific competitor; Cx = cortex; OB = olfactory bulbs and tubercles; Cb = cerebellum; St = striatum; Hc = hippocampus. All brain regions were analyzed on the same gel and under the same conditions to allow direct comparison between regions. **B**. The sum intensity of the first shifted band. OB&T = olfactory bulbs and the tubercles; Cb = cerebellum; Hippo = hippocampus.

**Figure 4:**
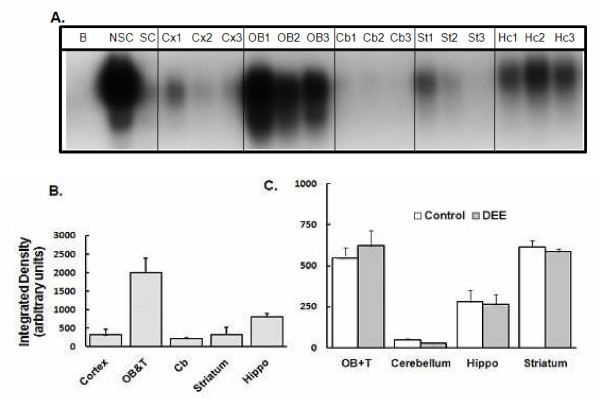
**AP1 activation**. **A**. A typical gel showing the AP-1 specific shifted band for three separate control samples (designated as 1, 2, or 3). B = Blank; SC = Specific competitor; NSC = Non-specific competitor; Cx = cortex; OB = olfactory bulbs and tubercles; Cb = cerebellum; St = striatum; Hc = hippocampus. All brain regions were analyzed on the same gel and under the same conditions to allow direct comparison between regions. **B**. The sum intensity of the shifted band. OB+T = olfactory bulbs and the tubercles; Cb = cerebellum; Hippo = hippocampus. **C**. The sum intensity of AP-1 specific shifted band in different regions of the rat brain after exposure to purified air (Control) or DEE (Diesel). Each brain region was analyzed on different days on separate gels and therefore this figure does not allow direct comparison between various brain regions. Bars represent mean of 5 individual animals ± SE.

The transcription factor NF-κB is an immunologically relevant transcription factor and upon activation, promotes the transcription of a variety of immunomodulatory factors including TNF-α. The levels of activated NF-κB were assessed in the nuclear fractions derived from the olfactory bulb and the tubercles (OB+T), cortex, striatum, hippocampus, and cerebellum. DEE exposure did not alter the levels of this transcription factor (Figure 2). When the basal levels of NF-κB were evaluated, there were evident regional variations (Figure 3). The cortex had the lowest while the OB+T showed the highest constitutive activity. The regional variation in baseline activity was more pronounced for AP-1 (Figure 4A). Again, the OB+T showed the highest while the cerebellum had the lowest basal activity (Figure 4B). DEE exposure did not alter the levels of AP-1 (Figure 4C) in any of the regions analyzed. It should be noted that the regional differences in the 'basal' levels of transcription factor activity may have been modulated by the ozone pretreatment conducted before the initiation of the DEE exposures.

### mRNA levels for TNF-α and TNF-RI

**Figure 5:**
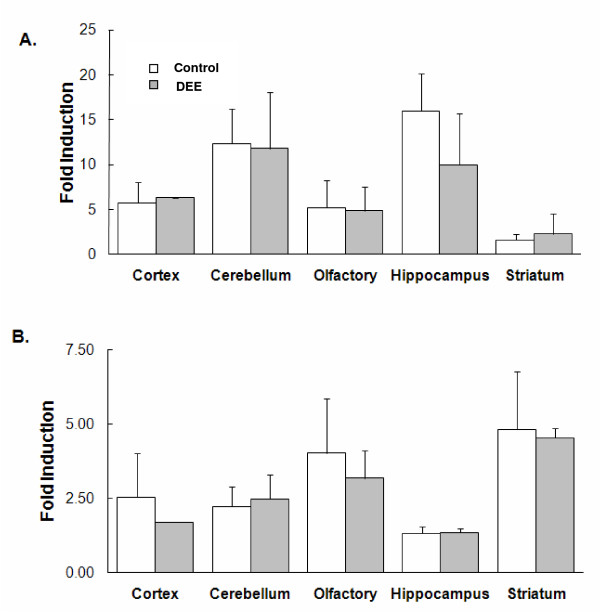
**RNA expression**. **A**. RNA expression for TNF-α. Bars represent mean of 5 individual animals SE. There are no error bars for the cortex of DEE-exposed animals because only one sample was available. **B**. RNA expression for TNF-RI. Bars represent mean of 5 individual animals ± SE. Bars represent mean of 5 individual animals SE. There are no error bars for the cortex of DEE-exposed animals because only one sample was available.

Since the TNF-α protein level was modified in specific brain regions after DEE exposures, the expression of mRNA levels for TNF-α was determined in various regions of rat brains. There was again a difference in basal expression and the striatum showed the lowest, while the hippocampus had the highest constitutive expression of the cytokine (Figure 5A). After exposure to diesel exhaust, there was no difference in mRNA levels for this proinflammatory cytokine in any of the brain regions analyzed. We also assessed the levels of TNF receptor subtype I (TNF-RI) in specific brain regions and exposure to DEE did not modify the levels of the receptor in any of the brain areas analyzed (Figure 5B).

## Discussion

Air pollution is a complex mixture of gases and particulate matter. DEE contributes substantially to combustion-derived nanoparticles which are an important component of air pollution [2]. In some urban areas, the air quality is so poor that the threshold considered 'safe' is consistently surpassed. In such environments, exposure to ambient air pollution and the possibility of adverse human health effects is a realistic cause for concern. While the connection between exposure to particulate matter and harmful cardiopulmonary effects has been reasonably well established [18,19], there is growing evidence that the CNS may be another target [5]. In the present study we demonstrate that prolonged exposure to moderate levels of DEE increased TNF-α and IL-1α protein levels specifically in the striatum of rat brains. However, none of the measured transcription factors (NF-κB and AP-1) or the mRNA levels of TNF-α and TNF-RI were affected after DEE exposure.

Numerous studies show that upregulation of markers associated with neuroinflammation may be either beneficial or harmful. For instance, TNF-α and TNF-RI have been shown to be involved in both neuroprotection [20] as well as neurodegeneration [21]. This dichotomy in TNF-α-related pathways may be attributed to the intensity [22] or CNS location [23] of neuroinflammation. The striatal dopaminergic system may be more vulnerable to adverse consequences of TNF-α production. As an example, in TNF receptor deficient mice, there is less striatal dopaminergic cell death induced by 1-methyl-4-phenyl-1,2,3,6-tetrahydropyridine (MPTP), a selective toxin for dopaminergic cells [24].

TNF-α levels are increased in the striatum and cerebrospinal fluid of Parkinson's disease patients when compared to controls [25]. Furthermore, several studies show that certain TNF-α polymorphisms in genotype or promoter sequences increase the risk for Parkinson's disease [26-28]. Our finding that exposure to DEE increases TNF-α selectively in the striatum may suggest that such environmental exposures may further aggravate factors associated with neurodegenerative disorders such as Parkinson's disease.

In the rat brain, the levels of transcription factor activity and cytokines were different depending on the region analyzed (Table 1). It is possible that the ozone pretreatment influenced this 'baseline' transcription factor activity. However, since the ozone exposure was conducted four weeks prior to tissue harvesting, it is likely that the ozone effects may be negligible at this time point. The region-specific activation state of transcription factors may be related to the unique cellular and molecular composition and function of different brain regions. For example, the olfactory bulb is a region where progenitor cells can give rise to new neurons [29] and this may underlie the high basal level of AP-1 and NF-κB activity detected. Indeed, it has been shown that a subunit of AP-1 (DeltaFosB) is upregulated after insult-induced neurogenesis and induces proliferation of neuronal precursor cells after injury [30]. NF-κB activity has been associated with neurogenesis and neuronal survival through anti-apoptotic gene expression although this transcription factor has also been implicated in neurodegeneration attributed to its proinflammatory characteristics. It appears that the intensity and cell type location of NF-κB activity determines its propensity to induce neuroprotection or neurotoxicity [31]. Although the protein levels of TNF-α and IL-1α were increased in the striatum of DEE-exposed rats, the levels of NF-κB activation (a factor which promotes transcription of immune-related genes) or TNF-α and TNF-RI RNA expression were unchanged. This may be due to homeostatic regulation of gene expression for the cytokines to protect against uncontrolled neuroinflammation. However, it may also be possible that the change in proinflammatory cytokine levels is mediated by systemic responses after exposure to DEE and the cytokines are entering the brain from peripheral sources. Exposure to particles derived from ambient air pollution increases inflammatory processes in the lungs [32,33] and intratracheal instillation of washed diesel exhaust particles can aggravate lung and systemic inflammation in mice [34]. Therefore, we cannot exclude that the proinflammatory cytokine changes in the CNS are due to systemic inflammation. However, we did not observe signs of increased inflammation in the lungs of the animals after DEE exposure (data not shown).

**Table 1:** Basal levels of transcription factor activation, mRNA expression, and protein levels in different regions of the rat brain.

Brain Regions	Transcription Factor		RNA		Protein	
	
	NF-κB	AP-1	TNF-α	TNFRI	TNFα	IL-1α
OB+T	+++	+++	+	+	+/++	++

Cortex	+	+	+	+	+	+

Striatum	+/++	+	+	+	++	++/+++

Hippocampus	++	++	+/++	+	++	++/+++

Cerebellum	++/+++	+	+/++	+	+/++	++/+++

For comparison purposes, values are designated as (+ = low; ++ = medium; or +++ = high) based on arbitrary assessment between groups for each marker.

The CNS effects reported in this study may be direct. The route of exposure to PM is via inhalation, and thus there is a potential for compounds to rapidly enter the brain through the cribriform plate of the ethmoid bone after deposition in the olfactory epithelium. This could then lead to direct activation of innate immune responses in the CNS. For inhaled manganese, the olfactory route has been shown to allow access of this metal into the brain [35,36]. It is also possible for inhaled particles to indirectly enter the brain through the circulation. In humans, inhaled radiolabeled particles in the ultrafine size range entered the systemic circulation and were detected in extrapulmonary organs [37]. The same scenario was observed in rats exposed to inhaled nano-sized silver particles [38]. The BBB is formed by tight junctions between endothelial cells comprising cerebral microvasculature. The stringent regulation imposed by the BBB prevents harmful factors present in the peripheral circulation from entering cerebral tissue. Exposure to TNF-α has been shown to increase the levels of the transporter protein P-glycoprotein which is thought to tighten the BBB by increasing the efflux of compounds out of the CNS [39]. Diesel exhaust particles dose-dependently upregulate P-glycoprotein levels and activity in isolated rat brain capillaries. This effect was dependent on TNF-α released after exposure to DEP [40]. Therefore, in our study, the increase in TNF-α detected in the striatum may be a protective effect to increase efflux of particulates from the CNS. We did not directly measure neuronal cell death and thus we cannot ascertain that the DEE-induced increase in these proinflammatory cytokines is harmful or protective.

The concept that gene-environment interactions play an important role in the causation and progression of chronic neurodegenerative diseases has received much attention in the past few years. Exposure to combustion-related compounds, present in air pollution, increases proinflammatory cytokines in the striatum of rats, and may be an environmental stress factor which contributes to neuronal cell death in this region. This may be especially important if genetic susceptibility factors are present. It has been shown that although cultured human microglial cells, derived from different individuals, show similar basal gene expression profiles (for many cytokines, chemokines, and growth factors) treatment with TNF-α leads to a completely different response depending on the individual source of the microglia [41]. Similar to this finding, it may be possible that there are individual differences in CNS inflammatory responses after exposure to DEE. Further studies are warranted before it can be concluded with certainty that prolonged exposure to components of air pollution may contribute to neuronal cell loss and whether genetic susceptibility factors may modulate this effect.

## Conclusion

In the present study we have shown that prolonged exposure to DEE induced a neuroinflammatory response in the rat brain in a region-specific manner. The inflammatory changes were assessed 24 hr post exposure and it is possible that this effect was transient. However, in a mouse model, neuroinflammatory markers were present two weeks after exposure to particulate matter [12] and thus it appears that the CNS effects are long-lasting although, species differences and PM sources need to be considered. To what extent the DEE-induced enhancement of inflammatory markers may lead to neurotoxicity or contribute to the progression of neurodegenerative diseases needs to be further evaluated and is the focus of our future research.

## Competing interests

The authors declare that they have no competing interests.

## Authors' contributions

MEGN has designed, coordinated and supervised the in vivo experimental work of this study, participated in the interpretation of the results and is co-writer of the manuscript. DvB participated in the mRNA analyses and interpretation of these data. FRC participated in conceiving the study, its design, interpretation of the results and is co-writer of the manuscript. RPFS participated in the mRNA analyses and interpretation of the results. KW participated in the ELISA, gel shift mobility assays and interpretation of the results. AC participated in the design and coordination of the study, processed the data including table and figures, carried out the statistical analysis, interpreted the results and drafted the manuscript. All authors have read, reviewed, commented and approved the final manuscript.
